# Aminomethylation/hydrogenolysis as an alternative to direct methylation of metalated isoquinolines – a novel total synthesis of the alkaloid 7-hydroxy-6-methoxy-1-methylisoquinoline

**DOI:** 10.3762/bjoc.14.8

**Published:** 2018-01-11

**Authors:** Benedikt C Melzer, Jan G Felber, Franz Bracher

**Affiliations:** 1 Department of Pharmacy – Center for Drug Research, Ludwig-Maximilians University of Munich, Butenandtstr. 5–13, D-81377 Munich, Germany

**Keywords:** alkaloid, aminomethylation, hydrogenolysis, isoquinoline, metalation, methylation

## Abstract

Highly-substituted isoquinolines are important scaffolds in syntheses of natural products and in drug development and hence, effective synthetic approaches are required. Here we present a novel method for the introduction of a methyl group at C1 of isoquinolines. This is exemplified by a new total synthesis of the alkaloid 7-hydroxy-6-methoxy-1-methylisoquinoline. Direct metalation of 7-benzyloxy-6-methoxyisoquinoline with Knochel–Hauser base, followed by cuprate-mediated methylation gives the target alkaloid directly, but separation from the educt is cumbersome. Quenching the metalated intermediate with Eschenmoser’s reagent gives an easy to clean tertiary benzylamine, which, after quaternization with iodomethane, is easily converted into the desired 1-methylisoquinoline by hydrogenolysis of both the benzylamine and benzyl ether groups.

## Introduction

The isoquinoline ring system has considerable relevance in drug development, since it is found in both complex [1] and “simple” alkaloids (variously substituted derivatives of the native isoquinoline) isolated from different plants [2], and antiviral and antimicrobial activities have been found for numerous isoquinoline alkaloids [3]. Further, isoquinoline is regarded as a “privileged scaffold” in drug design, and a large number of drug candidates containing this partial structure are in clinical development [4]. Consequently, synthetic approaches enabling a free variation of substituents on this heteroaromatic ring system are required. Numerous methods have been published over the decades for the construction of highly substituted isoquinolines [4]. Alternatively, subsequent functionalization of isoquinolines is feasible, e.g., via Pd-catalyzed C–H functionalization [5] or regioselective direct ring metalation (at C1) [6–8]. General aspects of the direct methylation of electron-deficient *N*-heterocycles have been reviewed [9].

**Figure 1:**
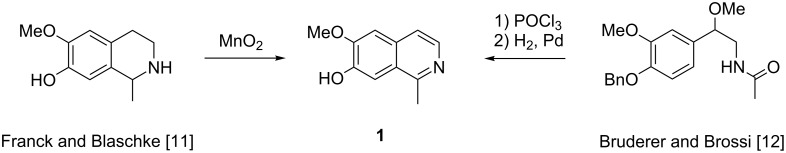
Previously published total syntheses of alkaloid **1** [11–12].

In a project aimed at the synthesis of tri- and tetracylic alkaloids containing the isoquinoline scaffold, we were interested in isoquinoline building blocks which bear a methyl group at C1, since this group should, due to its intrinsic C–H acidity, open opportunities for further functionalization. Since most isoquinoline alkaloids bear hydroxy and/or methoxy substituents at the positions 6 and 7, we regarded 7-hydroxy-6-methoxy-1-methylisoquinoline (**1**) as well as analogues containing a protected 7-hydroxy group as highly attractive building blocks for our project. A literature search revealed that compound **1** is in fact a natural product. It was isolated from the trunk bark of the Taiwanese tree *Hernandia nymphaeifolia* (Hernandiaceae) in 1996 [10]. No data on the biological activities of this alkaloid have been reported so far. Further, this literature search showed that two total syntheses of isoquinoline **1** had been published even before its identification as a natural product (Figure 1). In 1963, Franck and Blaschke [11] obtained **1** by dehydrogenation of its 1,2,3,4-tetrahydro analogue (which itself had to be prepared in several steps) with MnO_2_ in poor yield (24% crude product; yield of the final crystallization step for purification not given). In 1965, Bruderer and Brossi [12] reported on an approach whose central step was a Pictet–Gams cyclization of *N*-acetylated β,3-dimethoxy-4-benzyloxyphenethylamine, which gave the 7-*O*-benzyl derivative of alkaloid **1** in 27% yield. The required precursor is available from commercially available precursor **2** via a nitrostyrene intermediate in three steps in 45% overall yield [13]. In conclusion, no efficient approach to this “simple” alkaloid **1** has yet been published.

This prompted us to work out a novel synthetic access to alkaloid **1**. Our new strategy was fueled by our previous findings in the course of new approaches to benzylisoquinoline, oxoaporphine [7], and oxoisoaporphine alkaloids [8], where we could demonstrate the power of regioselective direct ring metalations of isoquinolines at C1 with sterically hindered amide bases like TMPMgCl∙LiCl (Knochel–Hauser base) [6]. For our present purpose, an appropriate 1-metalated isoquinoline species was to be converted into the corresponding 1-methyl product. Since the 7-hydroxy group of target alkaloid **1** is not compatible with the metalation reagent, the corresponding benzyl ether **3** was selected as central building block.

## Results and Discussion

**Scheme 1:**
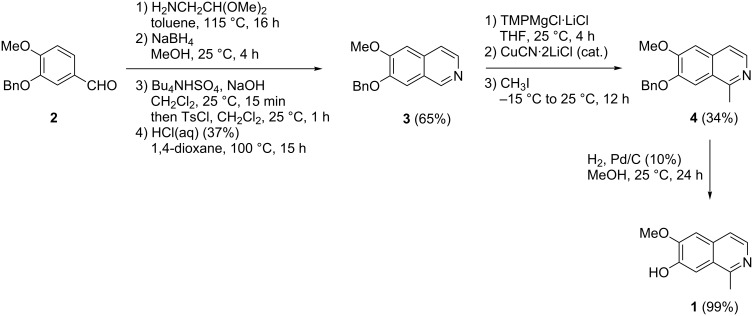
Total synthesis of alkaloid **1** via direct ring metalation and methylation.

In our previous work we prepared isoquinoline **3** in a three-step procedure starting from commercially available *O*-benzylisovanillin (**2**) in a modified Pomeranz–Fritsch reaction published by Reimann and Renz [14]. In the present investigations, we even optimized this procedure, ending up with a protocol that does not afford purification of any of the intermediates, and gives isoquinoline **3** in a straightforward operation in 65% isolated yield. For this purpose, the starting aldehyde **2** was subjected to a reductive amination with aminoacetaldehyde dimethyl acetal and NaBH_4_, followed by *N*-tosylation and hydrochloric acid-mediated cyclization under concomitant *N*-detosylation and aromatization. Direct ring metalation of **3** with TMPMgCl∙LiCl was performed as described by us previously [7]. First attempts for a direct methylation at C1 with iodomethane failed completely. Only upon addition of catalytic amounts of CuCN∙2LiCl [15] significant methylation took place. The crude reaction mixture contained almost equimolar amounts of the desired 1-methylisoquinoline **4** and starting material **3**. Due to the very similar polarities of **3** and **4**, chromatographic separation was very tedious, and only 34% of methyl compound **4** was isolated, accompanied by about 30% of starting material **3** and mixed fractions. Debenzylation of **4** by catalytic hydrogenation in methanol solution under palladium catalysis gave the alkaloid **1** in almost quantitative yield (Scheme 1).

So this new metalation/methylation protocol offered a new approach to alkaloid **1** with an overall yield (22% from commercially available precursor **2**) much higher than in the above mentioned previously published methods [11–12]. However, the unsatisfactory separation of 1-methylisoquinoline intermediate **4** from its unmethylated precursor **3** rendered this protocol unattractive. This is a problem that is common to numerous methylation protocols, including methylation of metalated arenes [16], methylation of arenes bearing directing groups with methylmagnesium bromide [17], and direct methylation using radical reactions [9,18–19].

Hence, we searched for a more viable method for the introduction of the methyl group. A prerequisite was that the organometallic intermediate should be trapped by an electrophile which could later be transformed into a methyl group, but the trapping product should be easily separable from the starting material **3**. For this purpose we selected Eschenmoser’s salt (*N*,*N*-dimethylmethyleniminium iodide) as an electrophile. Trapping of the metalated species was expected to give the *N*,*N*-dimethylaminomethyl derivative **5** in reasonable yield. Later on, hydrogenolytic cleavage of the generated benzylamine-type group at C1 should give the desired 1-methyl moiety. Related reductive cleavage reactions have been published earlier by Möhrle [20] for phenolic Mannich bases in the course of the total synthesis of the naphthalene-derived natural product plumbagin.

In fact, trapping 1-metalated isoquinoline **3** with Eschenmoser’s salt gave the aminomethyl derivative **5** in 37% yield. Chromatographic separation from starting material **3** (recovered yield: 32%) was unproblematic.

**Scheme 2:**
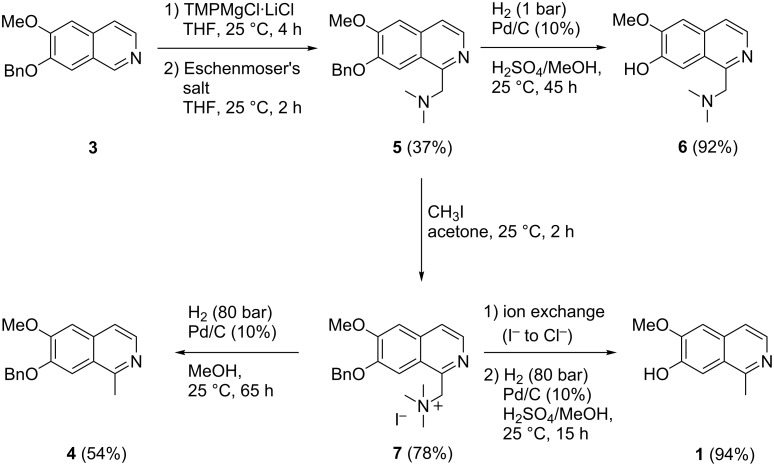
Total synthesis of alkaloid **1** via the aminomethyl intermediate **5** and selectivity’s found in debenzylation experiments.

Surprising results were obtained in our hydrogenolysis experiments with **5**, which were aimed at simultaneous *O*-debenzylation at the 7-position and conversion of the *N*,*N*-dimethylaminomethyl group at C1 into a methyl group. Hydrogenation in presence of palladium as catalyst at 1 bar in the presence or absence of small amounts of sulfuric acid gave the phenolic product **6** in high yield with unchanged *N*,*N*-dimethylaminomethyl group. The same result was obtained at high pressure (40 bar) and upon addition of formic acid for accelerating hydrogenolysis [21]. Obviously, and in contrast to earlier reports on related naphthol Mannich bases [20], the benzylamine moiety of **5** is resistant to hydrogenolysis, whereas the benzyl ether is readily removed. This order of reactivity is known from previous work [22]. Since it is further known that quaternary benzylammonium compounds undergo hydrogenolysis easier than the corresponding tertiary benzylamines [22], we converted amine **5** into methoiodide **7** by treatment with iodomethane. This salt was obtained in pure form in 78% yield by simply collecting the precipitate, and there was no indication of an undesired methylation of the ring nitrogen of the isoquinoline. High-pressure hydrogenolysis of **7** gave only minor amounts (about 7%) of the desired alkaloid **1**, but 1-methyl compound **4** with intact *O*-benzyl residue was isolated in 54% yield. The surprising stability of the *O*-benzyl residue in this experiment might be due to the iodide counterion, which is known to be a poison for palladium catalysts. Cleavage of the highly reactive benzylammonium residue still takes place, but *O*-debenzylation is predominantly suppressed by this catalyst poison. Finally, poisoning of the catalyst was prevented by simply passing a solution of the methoiodide **7** through a chloride-loaded ion exchanger prior to catalytic hydrogenation. In this manner alkaloid **1** was obtained in 94% yield (18% overall yield from commercially available precursor **2**, Scheme 2).

## Conclusion

In conclusion, we have worked out two novel protocols for the introduction of a methyl group at C1 of isoquinolines. These were applied to the total synthesis of the alkaloid 7-hydroxy-6-methoxy-1-methylisoquinoline (**1**), but should also be of value for the synthesis of other 1-methylisoquinolines. We could demonstrate that the aminomethylation of metalated arenes with Eschenmoser’s salt followed by hydrogenolytic cleavage is a highly attractive alternative to direct ring methylations. Further, new insights into selectivity of *O*- and *N*-debenzylation reactions should be useful for future natural product and drug syntheses.

## Experimental

For experimental procedures and copies of ^1^H and ^13^C NMR spectra of all compounds see Supporting Information File 1.

## Supporting Information

File 1 Experimental part and NMR spectra.
