# Supplementary material for: Aminomethylation/hydrogenolysis as an alternative to direct methylation of metalated isoquinolines – a novel total synthesis of the alkaloid 7-hydroxy-6-methoxy-1-methylisoquinoline
Source: Beilstein J Org Chem. 2018 Jan 11;14:130–4. doi: 10.3762/bjoc.14.8 (PMC5789437; doi:10.3762/bjoc.14.8)
Supplement: File 1 — Experimental part and NMR spectra. [file Beilstein_J_Org_Chem-14-130-s001.pdf]

Supporting Information

for

Aminomethylation/hydrogenolysis as an alternative to direct methylation of metalated isoquinolines – a novel total synthesis of the alkaloid 7-hydroxy-6-methoxy-1-methylisoquinoline

Benedikt C. Melzer, Jan G. Felber and Franz Bracher*

Address: Department of Pharmacy – Center for Drug Research, Ludwig-Maximilians University of Munich, Butenandtstr. 5–13, D-81377 Munich, Germany

Email: Franz Bracher* - Franz.Bracher@cup.uni-muenchen.de

*Corresponding author

Experimental par and NMR spectra

Table of contents

Experimental procedures and characterization of all compounds ………………………. S2–8
^{1}H NMR spectrum of **3**……………………………………………………………….. S9
^{13}C NMR spectrum of **3**……………………………………………………………… S10
^{1}H NMR spectrum of **4**……………………………………………………………….. S11
^{13}C NMR spectrum of **4**……………………………………………………………… S12
^{1}H NMR spectrum of **5**……………………………………………………………….. S13
^{13}C NMR spectrum of **5**……………………………………………………………… S14
^{1}H NMR spectrum of **6**………………………………………………………………. S15
^{13}C NMR spectrum of **6**……………………………………………………………….. S16
^{1}H NMR spectrum of **7**……………………………………………………………… S17
^{13}C NMR spectrum of **7**……………………………………………………………….. S18
^{1}H NMR spectrum of **1**……………………………………………………………… S19
^{13}C NMR spectrum of **1**……………………………………………………………… S20
References……………………………………………………………………………… S21

Experimental procedures and characterization of all compounds

General Information

Solvents used were of HPLC grade or p.a. grade and/or purified according to standard procedures. Melting points were determined by open tube capillary method with a Büchi melting point B-450 apparatus. IR measurements were carried out with a Perkin–Elmer FTIR Paragon 1000 spectrometer as KBr pellets or with a Jasco FT/IR-4100 with ATR PRO450-S corrected to KBr. NMR spectra were recorded with Jeol J NMR GX (400 or 500 MHz) and Avance III HD Bruker BioSpin (400 or 500 MHz) spectrometers with residual non-deuterated solvent as internal standard. Spectra were recorded in deuterated solvents and chemical shifts are reported in parts per million (ppm). *J* values are given in Hertz. Multiplicities are abbreviated as follows: s = singlet, d = doublet, t = triplet, m = multiplet. Signal assignments were carried out based on ^{1}H, ^{13}C, HMBC, HMQC and COSY spectra. NMR spectra were analyzed with the NMR software MestReNova, Version 5.1.1-3092 (Mestrelab Research S.L.). HRMS were performed by electron impact (EI) at 70 eV with a Thermo Finnigan MAT 95 or a Jeol GCmate II spectrometer or by electrospray ionization (ESI) with a Thermo Finnigan LTQ FT Ultra Fourier Transform Ion Cyclotron resonance mass spectrometer. Chromatographic purification of products was performed by using flash column chromatography on Merck silica gel 60 (0.015–0.040 mm) as stationary phase.

7-Benzyloxy-6-methoxyisoquinoline (3)

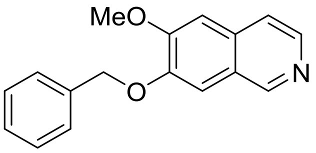

3-Benzyloxy-4-methoxybenzaldehyde (**2**, 2.22 g, 9.16 mmol) was dissolved in toluene (50 mL), before aminoacetaldehyde dimethylacetal (1.06 g, 10.08 mmol) was added. The reaction mixture was heated to reflux (115 °C) for 15 h. After cooling to room temperature the solvent was removed under reduced pressure. The residue was suspended in

methanol (50 mL), cooled to 0 °C and sodium borohydride (0.693 g, 18.32 mmol) was added portionwise to give a clear solution. The resulting mixture was stirred at 25 °C for 4 h. Then the solvent was removed under reduced pressure. Water (50 mL) was added and the mixture was extracted with dichloromethane (3 × 50 mL). The combined organic layers were dried over Na_2SO_4 and concentrated under reduced pressure. The residue was dissolved in dichloromethane (50 mL), before sodium hydroxide (0.623 g, 15.6 mmol) and tetrabutylammonium hydrogensulfate (0.218 g, 0.641 mmol) were added. After stirring at 25 °C for 15 min a solution of *p*-toluene sulfonylchloride (2.10 g, 11.0 mmol) in dichloromethane (35 mL) was added dropwise over 1 h. After complete addition the reaction mixture was stirred at 25 °C for 1 h. Then water (50 mL) was added and the organic layer was separated, washed with water (2 × 50 mL) and brine (50 mL), dried over Na_2SO_4 and concentrated under reduced pressure. The residue was dissolved in 1,4-dioxane (80 mL) and an 6 M aqueous solution of HCl (15 mL) was added. The reaction mixture was heated to reflux for 15 h. After cooling to room temperature, the mixture was diluted with water (80 mL), washed with diethyl ether (2 × 50 mL), then basified with 6 M NaOH (pH > 9) and extracted with dichloromethane (3 × 100 mL). The combined organic layers were dried over Na_2SO_4 and concentrated under reduced pressure. The residue was purified by flash column chromatography (ethyl acetate) to give **3** as white solid (1.58 g, 65%). mp 152 °C (lit. [1] 150 – 153 °C); ^{1}H NMR (400 MHz, $CDCl_3$): δ (ppm) = 8.98 (s, 1H), 8.38 (d, *J* = 5.7 Hz, 1H), 7.54 – 7.46 (m, 3H), 7.44 – 7.37 (m, 2H), 7.37 – 7.31 (m, 1H), 7.24 (s, 1H), 7.08 (s, 1H), 5.29 (s, 2H), 4.03 (s, 3H); ^{13}C NMR (101 MHz, $CDCl_3$): δ (ppm) = 153.4, 150.1, 149.4, 142.1, 136.2, 132.6, 128.7, 128.2, 127.4, 124.6, 119.2, 107.4, 104.8, 70.8, 56.1; HRMS (EI): *m/z* (%) = 265.1098 $[M]^+$ (calcd for $C_{17}H_{15}NO_2$: 265.1097); IR (ATR): ν (cm^{-1}) = 1619, 1571, 1503, 1481, 1456, 1378, 1333, 1255, 1248, 1223, 1201, 1141, 987, 932, 923, 875, 857, 746, 697, 596, 579.

7-Benzyloxy-6-methoxy-1-methylisoquinoline (4)

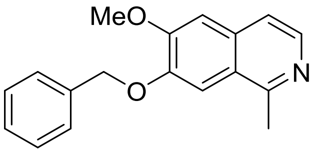

Method A: A dry and nitrogen flushed 50 mL Schlenk flask, equipped with a magnetic stirring bar, was charged with TMPMgCl·LiCl (1.0 M in THF/toluene; 3.0 mL, 3.00 mmol). A solution of 7-benzyloxy-6-methoxyisoquinoline (**3**, 0.531 g, 2.00 mmol) in dry THF (10 mL) was added and the reaction mixture was stirred at 25 °C for 4 h. After cooling to −15 °C CuCN·2LiCl (1.0 M in THF; 0.04 mL, 0.040 mmol) was added directly followed by methyl iodide (0.426 g, 3.00 mmol). The mixture was allowed to warm to 25 °C within 12 h. Then the mixture was quenched with satd. aqueous NH_4Cl solution (8 mL) and extracted with dichloromethane (4 × 30 mL). The combined organic layers were dried over Na_2SO_4 and concentrated under reduced pressure. The residue was purified by flash column chromatography (gradient: 25% ethyl acetate in dichloromethane to 100% ethyl acetate) to give **4** as a white solid (0.188 g, 34%).

Method B: To a suspension of **7** (0.030 g, 0.064 mmol) in methanol (5 mL) was added 10% Pd/C (0.018 g). The mixture was stirred under hydrogen atmosphere (80 bar) at 25 °C for 65 h. Then the catalyst was filtered off through a pad of celite and the resulting solution was concentrated under reduced pressure. The residue was purified by flash column chromatography (dichlorometane/methanol 9:1) to give **4** as a white solid (0.010 g, 54%). mp 162 °C (lit. [2] 162 °C); ^{1}H NMR (400 MHz, $CDCl_3$): δ (ppm) = 8.25 (d, *J* = 5.7 Hz, 1H), 7.54 – 7.50 (m, 2H), 7.44 – 7.38 (m, 2H), 7.38 – 7.29 (m, 3H), 7.07 (s, 1H), 5.30 (s, 2H), 4.02 (s, 3H), 2.80 (s, 3H); ^{13}C NMR (126 MHz, $CDCl_3$): δ (ppm) = 155.9, 153.1, 148.8, 140.7, 136.3, 132.8, 128.7 (2C), 128.2, 127.5 (2C), 123.1, 118.2, 106.4, 105.4, 71.0, 56.0, 22.3; HRMS (EI): *m/z* (%) = 279.1254 $[M]^+$ (calcd for $C_{18}H_{17}NO_2$: 279.1259); IR (KBr pellet): *v* (cm^{-1}) = 1616, 1567, 1503, 1479, 1432, 1371, 1272, 1227, 1196, 1160, 1057, 983, 919, 863, 857, 757, 702, 597, 587.

1-(7-Benzyloxy-6-methoxyisoquinolin-1-yl)-*N*,*N*-dimethylmethanamine (5)

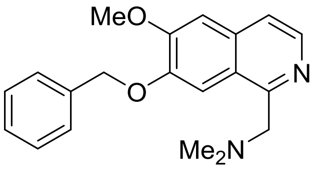

A dry and nitrogen flushed 50 mL Schlenk flask, equipped with a magnetic stirring bar, was charged with TMPMgCl·LiCl (1.0 M in THF/toluene; 3.0 mL, 3.00 mmol). A solution of **3** (0.531 g, 2.00 mmol) in dry THF (10 mL) was added and the reaction mixture was stirred at 25 °C for 4 h. After cooling to 0 °C neat Eschenmoser's salt (*N*,*N*-dimethylmethylenammonium iodide) (0.555 g, 3.00 mmol) was added under nitrogen counter flow and the mixture was stirred at 25 °C for 2 h. Then the reaction was quenched by with satd. aqueous $NaHCO_3$ solution (5 mL) and water (5 mL) and was extracted with dichloromethane (3 × 30 mL). The combined organic layers were dried over Na_2SO_4 and concentrated under reduced pressure. The residue was purified by flash column chromatography (gradient: 3% triethylamine in ethyl acetate to 3% triethylamine + 5% methanol in ethyl acetate) to give **5** as a white solid (0.235 g, 37%). mp 127 °C; ^{1}H NMR (400 MHz, $CDCl_3$): δ (ppm) = 8.29 (dd, *J* = 5.6, 0.7 Hz, 1H), 7.84 (d, *J* = 0.7 Hz, 1H), 7.55 – 7.48 (m, 2H), 7.43 – 7.35 (m, 3H), 7.35 – 7.29 (m, 1H), 7.06 (s, 1H), 5.33 (s, 2H), 4.02 (s, 3H), 3.83 (s, 2H), 2.25 (s, 6H); ^{13}C NMR (101 MHz, $CDCl_3$): δ (ppm) = 156.1, 153.1, 148.7, 140.8, 136.6, 133.5, 128.8 (2C), 128.2, 127.6 (2C), 123.7, 119.3, 107.1, 105.2, 70.9, 65.2, 56.1, 45.8 (2C); HRMS (ESI): *m/z* (%) = 323.1753 $[M+H]^+$ (calcd for $C_{20}H_{23}N_2O_2^+$: 323.1754); IR (ATR): *v* (cm^{-1}) = 2958, 1739, 1615, 1566, 1503, 1477, 1468, 1435, 1390, 1377, 1281, 1253, 1227, 1198, 1159, 1144, 1054, 1009, 985, 858, 831, 700, 651, 600, 582.

1-[(Dimethylamino)methyl]-6-methoxyisoquinolin-7-ol (6)

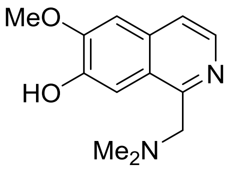

To a solution of **5** (0.182 g, 0.560 mmol) in methanol (5 mL) was added 10% Pd/C (0.018 g) and concentrated sulfuric acid (2 drops). The mixture was stirred under hydrogen atmosphere at 25 °C for 45 h. Then the catalyst was filtered off through a pad of celite and the resulting solution was concentrated under reduced pressure. The residue was dissolved with satd. aqueous $NaHCO_3$ solution (2 mL) and was extracted with dichloromethane (3 × 10 mL). The combined organic layers were dried over Na_2SO_4 and concentrated under reduced pressure to give almost pure **6** (0.130 g, 92%, corrected on basis of impurities detected by ^{1}H NMR) as a white solid without further purification. ^{1}H NMR (500 MHz, $CDCl_3$): δ (ppm) = 8.19 (d, *J* = 5.7 Hz, 1H), 7.60 (s, 1H), 7.34 (d, *J* = 5.7 Hz, 1H), 6.73 (s, 1H), 3.87 (s, 2H), 3.61 (s, 3H), 2.29 (s, 6H); ^{13}C NMR (126 MHz, $CDCl_3$): δ (ppm) = 154.6, 152.2, 148.0, 138.8, 132.7, 124.0, 119.7, 108.9, 104.3, 63.2, 55.4, 45.7 (2C); HRMS (ESI): *m/z* (%) = 233.1284 $[M+H]^+$ (calcd for $C_{13}H_{17}N_2O_2^+$: 233.1285).

1-(7-Benzyloxy-6-methoxyisoquinolin-1-yl)-*N,N,N*-trimethylmethanaminium iodide (7)

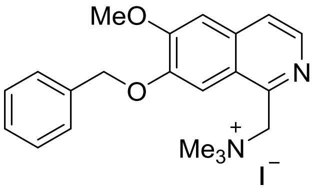

To a solution of **5** (0.074 g, 0.230 mmol) in acetone (5 mL) was added methyl iodide (0.036 g, 0.250 mmol) dropwise. The reaction mixture was stirred at 25 °C for 2 h and the precipitated white solid was filtered off, then dissolved in methanol (10.0 mL) and concentrated under reduced pressure to give **7** (0.083 g, 78%) as a white solid without further purification. mp 211 °C; ^{1}H NMR (500 MHz, $(CD_3)_2SO$): δ (ppm) = 8.48 (d, *J* = 5.5 Hz, 1H), 7.85 (d, *J* = 5.5 Hz, 1H), 7.80 (s, 1H), 7.57 – 7.50 (m, 3H), 7.47 – 7.42 (m, 2H), 7.41 –

7.36 (m, 1H), 5.36 (s, 2H), 5.10 (s, 2H), 3.96 (s, 3H), 3.17 (s, 9H); ^{13}C NMR (126 MHz, $(CD_3)_2SO$): δ (ppm) = 153.0, 150.0, 146.4, 140.6, 136.3, 133.7, 128.6 (2C), 128.2 (3C), 124.6, 121.3, 106.0, 104.6, 70.6, 64.8, 56.0, 53.0 (3C); HRMS (ESI): *m/z* (%) = 337.1909 $[M+H]^+$ (calcd for $C_{21}H_{25}N_2O_2^+$: 337.1911); IR (ATR): *v* (cm^{-1}) = 1624, 1566, 1510, 1485, 1439, 1395, 1340, 1269, 1246, 1165, 1081, 1049, 1028, 998, 977, 905, 851, 746, 701, 652, 596 587.

6-Methoxy-1-methylisoquinolin-7-ol (1)

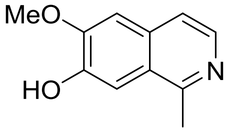

Method A: To a solution of **4** (0.150 g, 0.537 mmol) in methanol (25 mL) was added 10% Pd/C (0.100 g). The mixture was stirred under hydrogen atmosphere at 25 °C for 24 h. Then the catalyst was filtered off through a pad of celite and the resulting solution was concentrated under reduced pressure. The residue was purified by flash column chromatography (ethyl acetate/methanol = 95 : 5) to give **1** as a white solid (0.101 g, 99%).

Method B: A solution of **7** (0.050 g, 0.108 mmol) in methanol was passed through a chloride-saturated ion exchange column (ion exchanger III, Merck (Darmstadt, Germany); strongly basic anion exchanger, OH^- form, saturated with NaCl solution). To the eluted crude chloride salt, dissolved in methanol (5 mL), was added 10% Pd/C (0.018 g) and concentrated sulfuric acid (2 drops). The mixture was stirred under hydrogen atmosphere (80 bar) at 25 °C for 15 h. Then the catalyst was filtered off through a pad of celite and the resulting solution was concentrated under reduced pressure. The residue was dissolved in dichloromethane (5 mL) and washed with water (3 × 5 mL), dried over Na_2SO_4 and concentrated under reduced pressure to give **1** as a white solid (0.019 g, 94%).

mp 208 – 209 °C (lit. [3] 218 °C); ^{1}H NMR (400 MHz, $CDCl_3$): δ (ppm) = 8.23 (d, *J* = 5.8 Hz, 1H), 7.49 (s, 1H), 7.39 (d, *J* = 5.8 Hz, 1H), 7.07 (s, 1H), 4.04 (s, 3H), 2.84 (s, 3H); ^{13}C NMR (126 MHz, $CDCl_3$): δ (ppm) = 155.9, 151.2, 147.0, 139.3, 132.3, 123.8, 118.6, 107.6, 104.8, 56.1, 21.9; HRMS (EI): *m/z* (%) = 189.0766 $[M]^+$ (calcd for $C_{11}H_{11}NO_2$: 189.0790); IR (KBr

pellet): *v* (cm^{-1}) = 2998, 1626, 1503, 1477, 1434, 1365, 1338, 1270, 1228, 1194, 1159, 1061, 975, 855, 828, 663, 589.

Copies of ^{1}H and ^{13}C NMR spectra of all compounds

^{1}H NMR spectrum of 7-benzyloxy-6-methoxyisoquinoline (3)

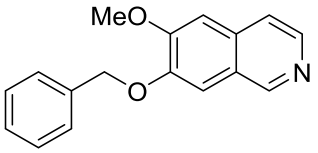

Frequency: 400 MHz

Solvent: $CDCl_3$

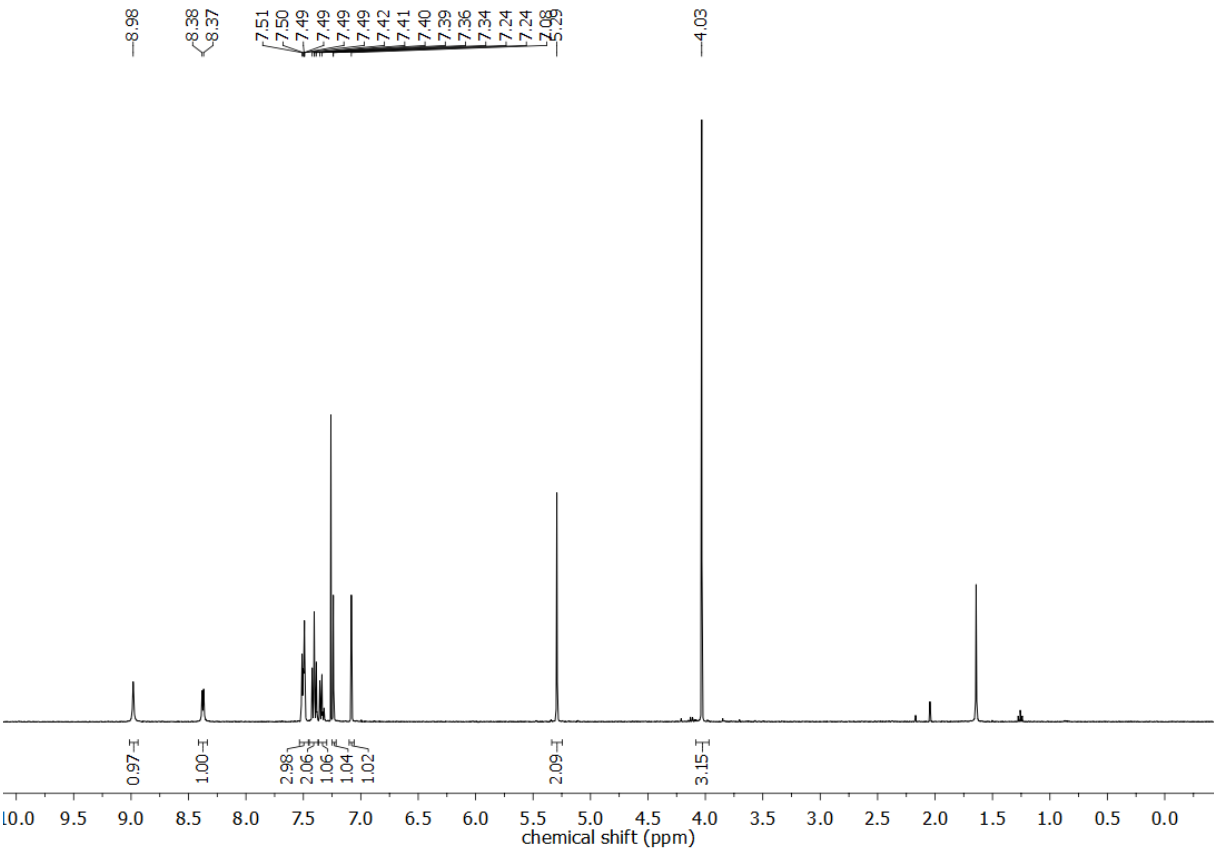

^{13}C NMR spectrum of 7-benzyloxy-6-methoxyisoquinoline (3)

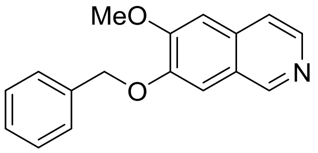

Frequency: 101 MHz

Solvent: $CDCl_3$

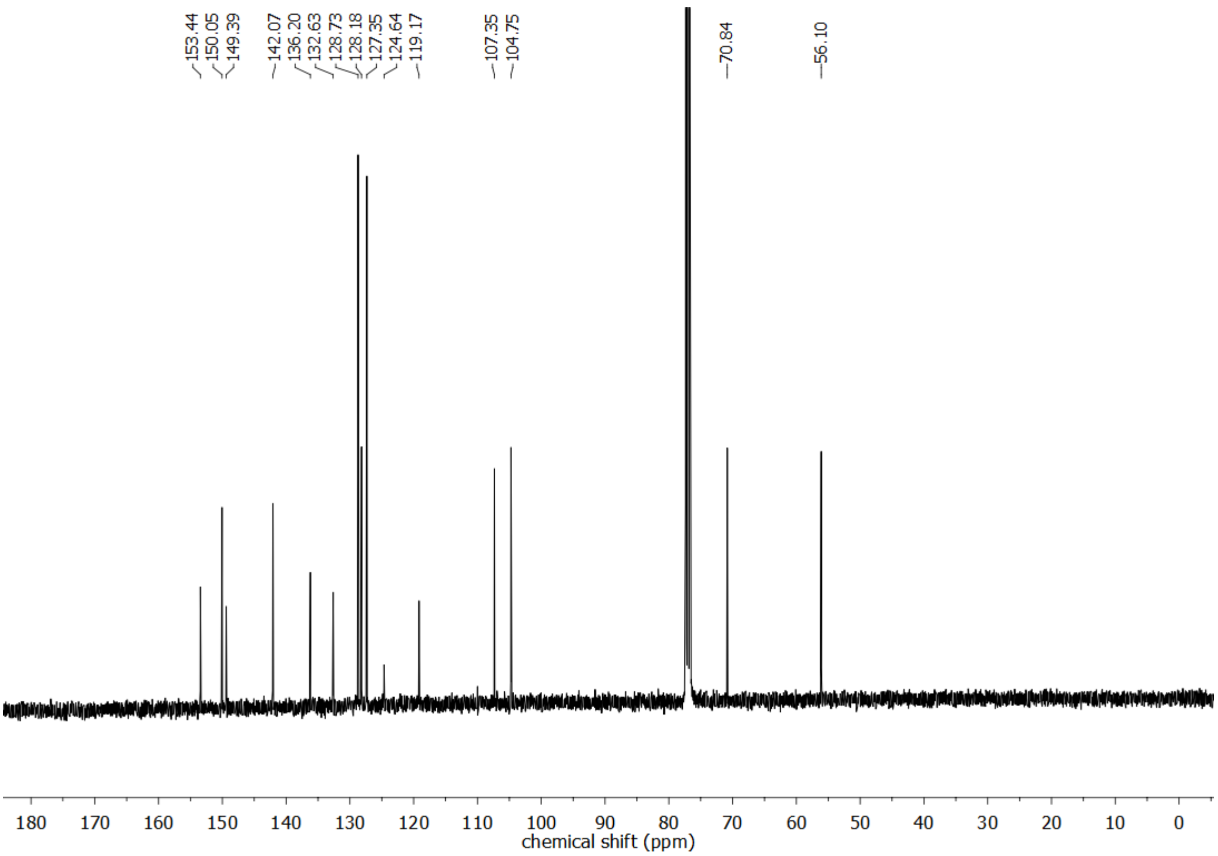

^{1}H NMR spectrum of 7-benzyloxy-6-methoxy-1-methylisoquinoline (4)

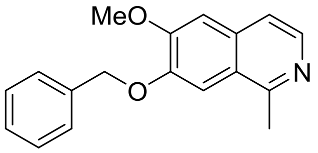

Frequency: 400 MHz

Solvent: $CDCl_3$

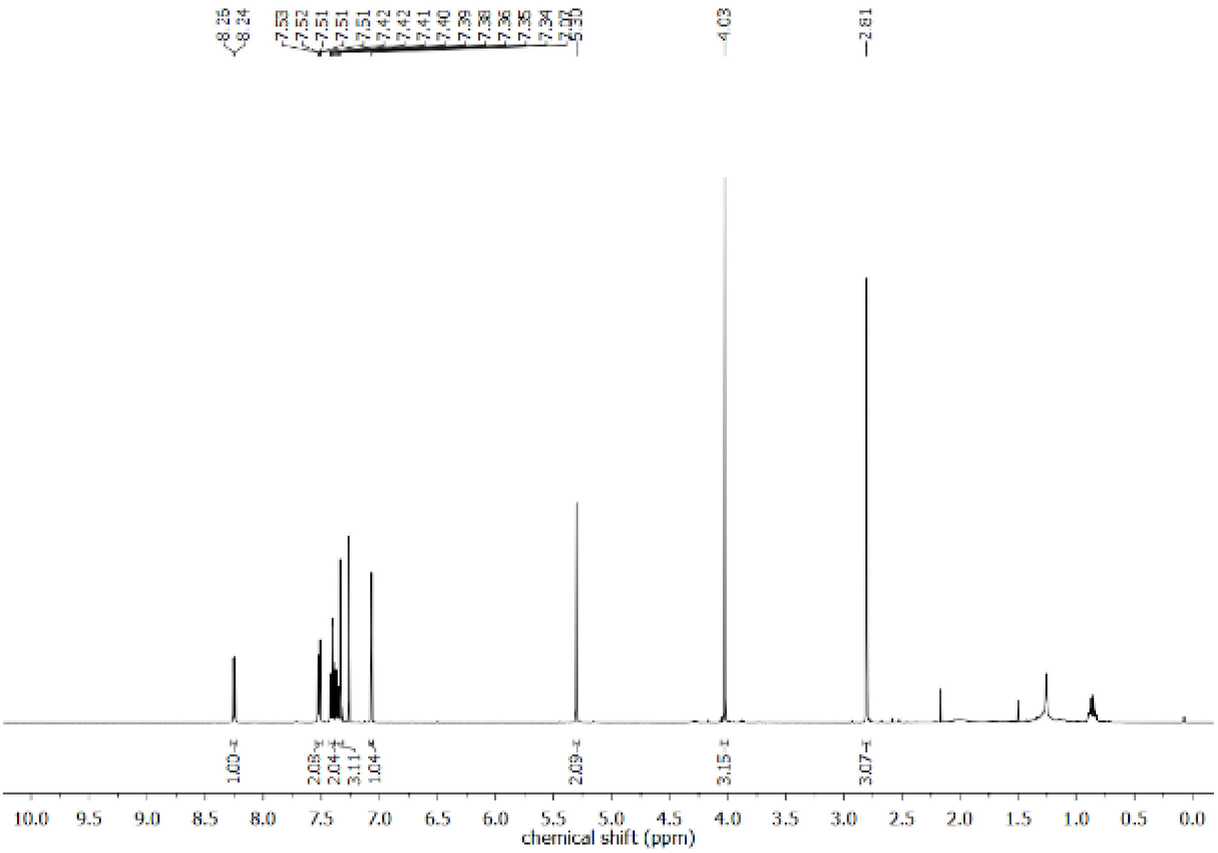

^{13}C NMR spectrum of 7-benzyloxy-6-methoxy-1-methylisoquinoline (4)

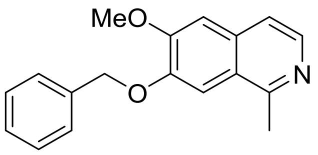

Frequency: 126 MHz

Solvent: $CDCl_3$

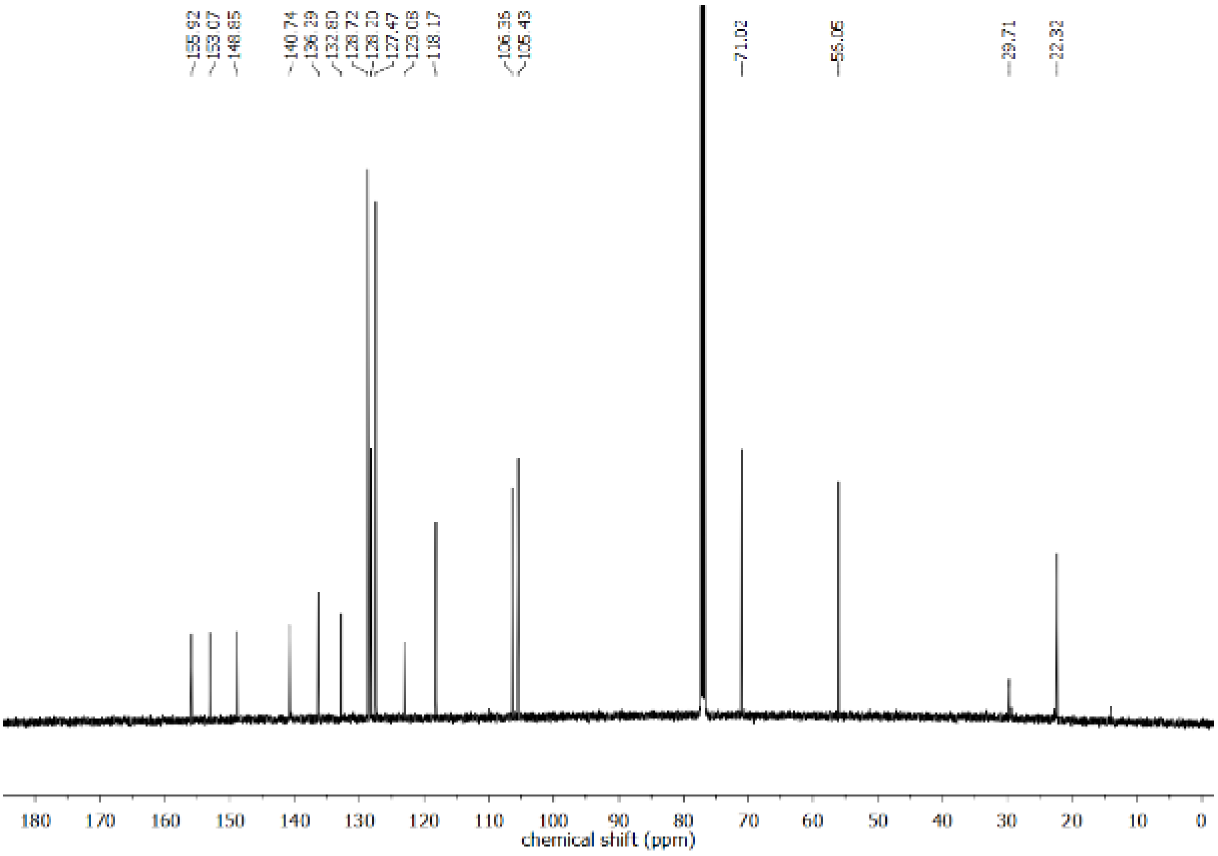

^{1}H NMR spectrum of 1-(7-benzyloxy-6-methoxyisoquinolin-1-yl)-*N*,*N*-dimethylmethanamine (5)

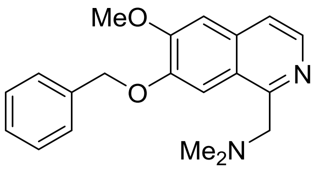

Frequency: 400 MHz

Solvent: $CDCl_3$

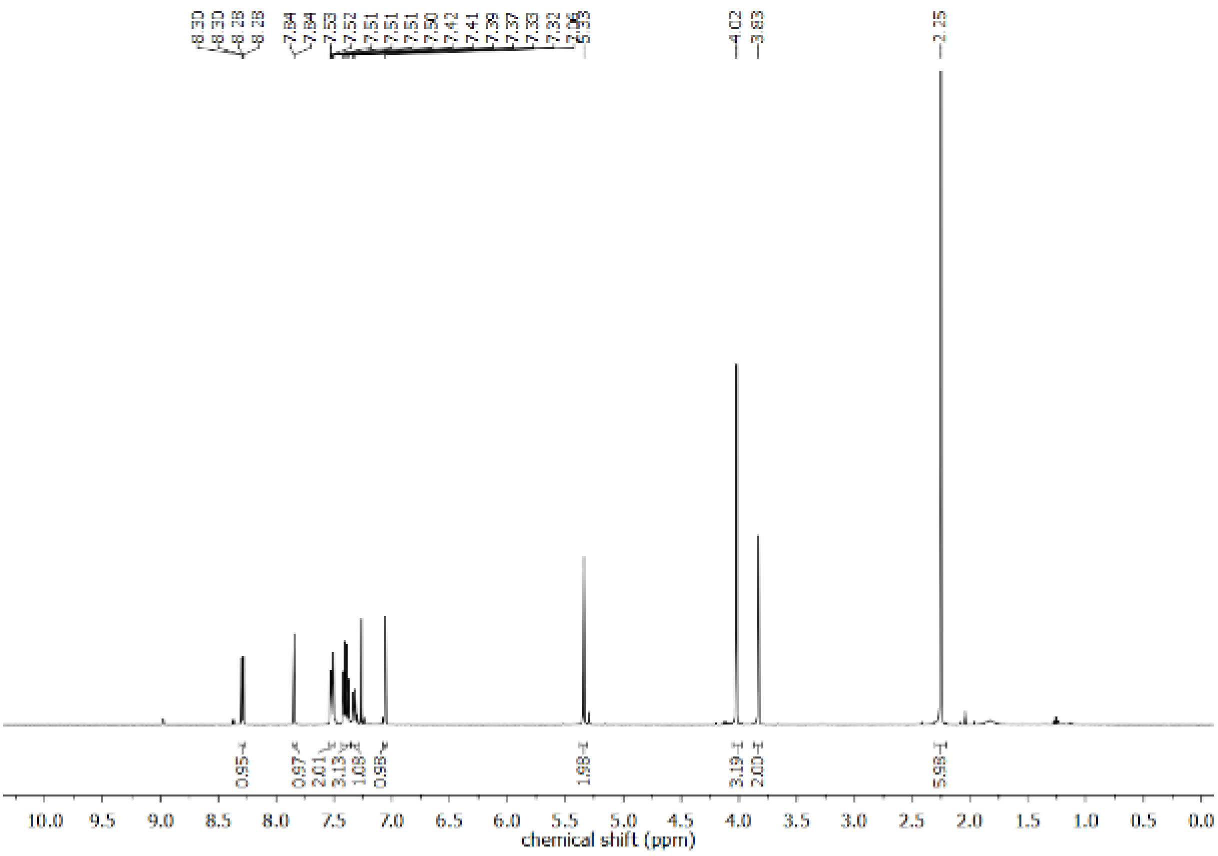

^{13}C NMR spectrum of 1-(7-benzyloxy-6-methoxyisoquinolin-1-yl)-*N*,*N*-dimethylmethanamine (5)

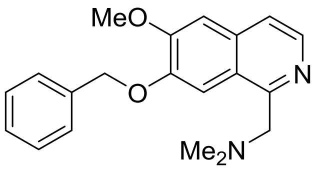

Frequency: 101 MHz

Solvent: $CDCl_3$

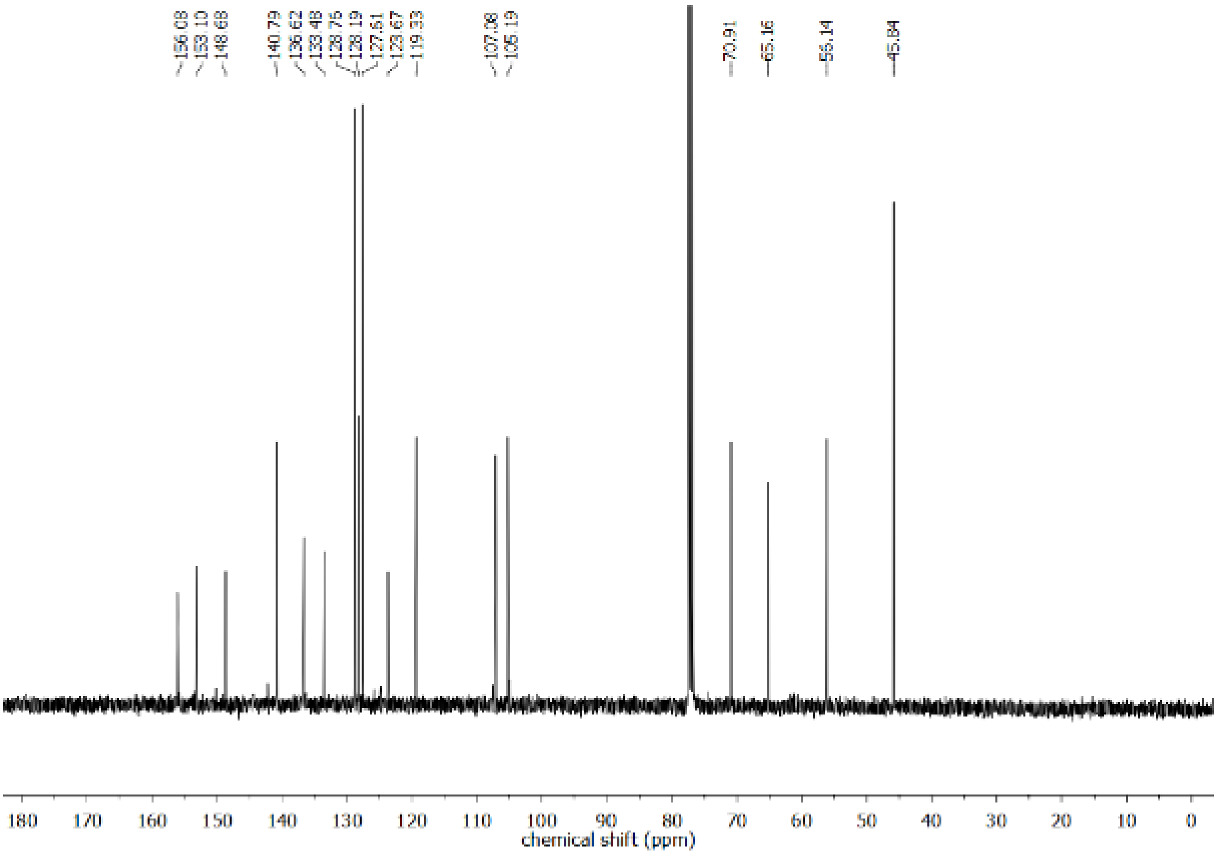

^{1}H NMR spectrum of 1-[(dimethylamino)methyl]-6-methoxyisoquinolin-7-ol (6)

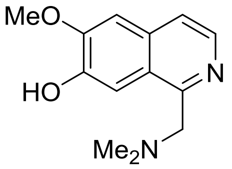

Frequency: 500 MHz

Solvent: $CDCl_3$

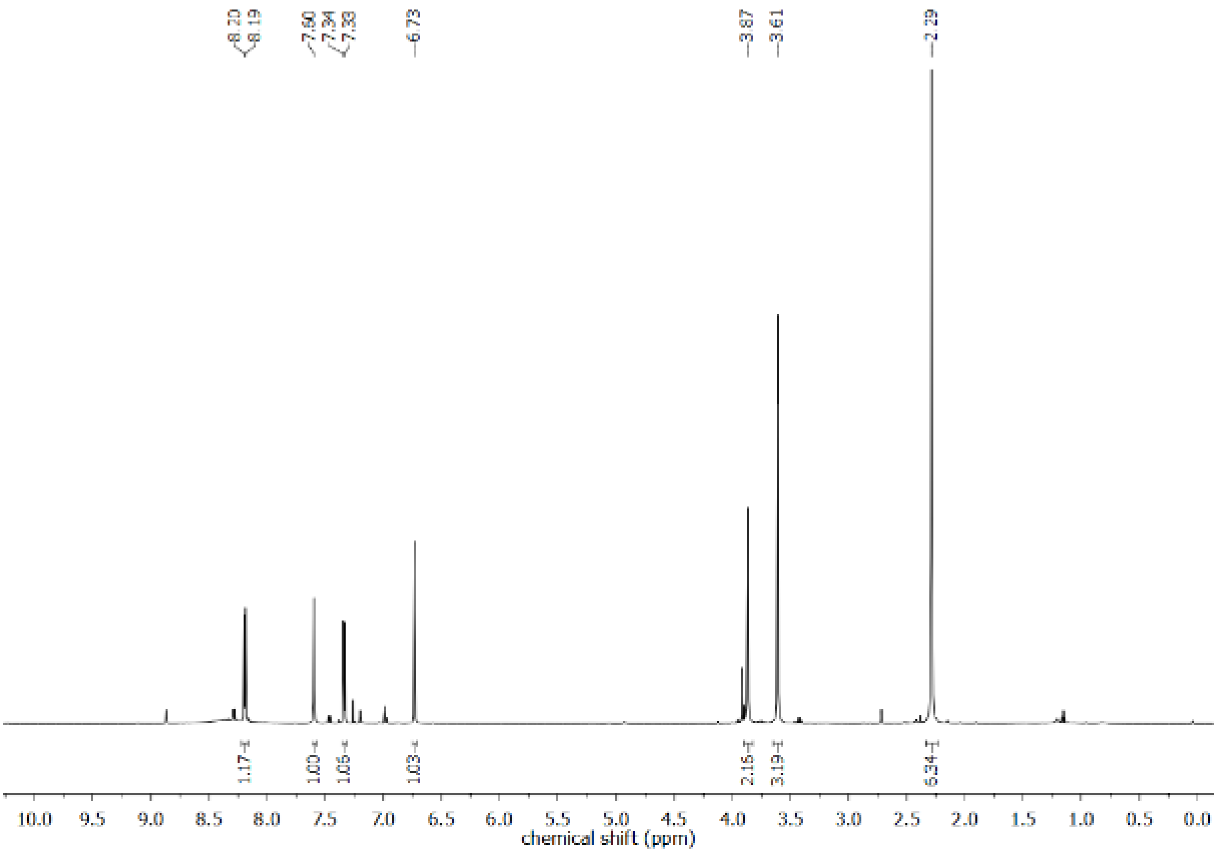

^{13}C NMR spectrum of 1-[(dimethylamino)methyl]-6-methoxyisoquinolin-7-ol (6)

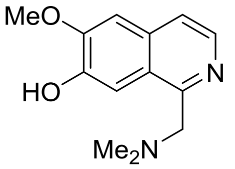

Frequency: 126 MHz

Solvent: $CDCl_3$

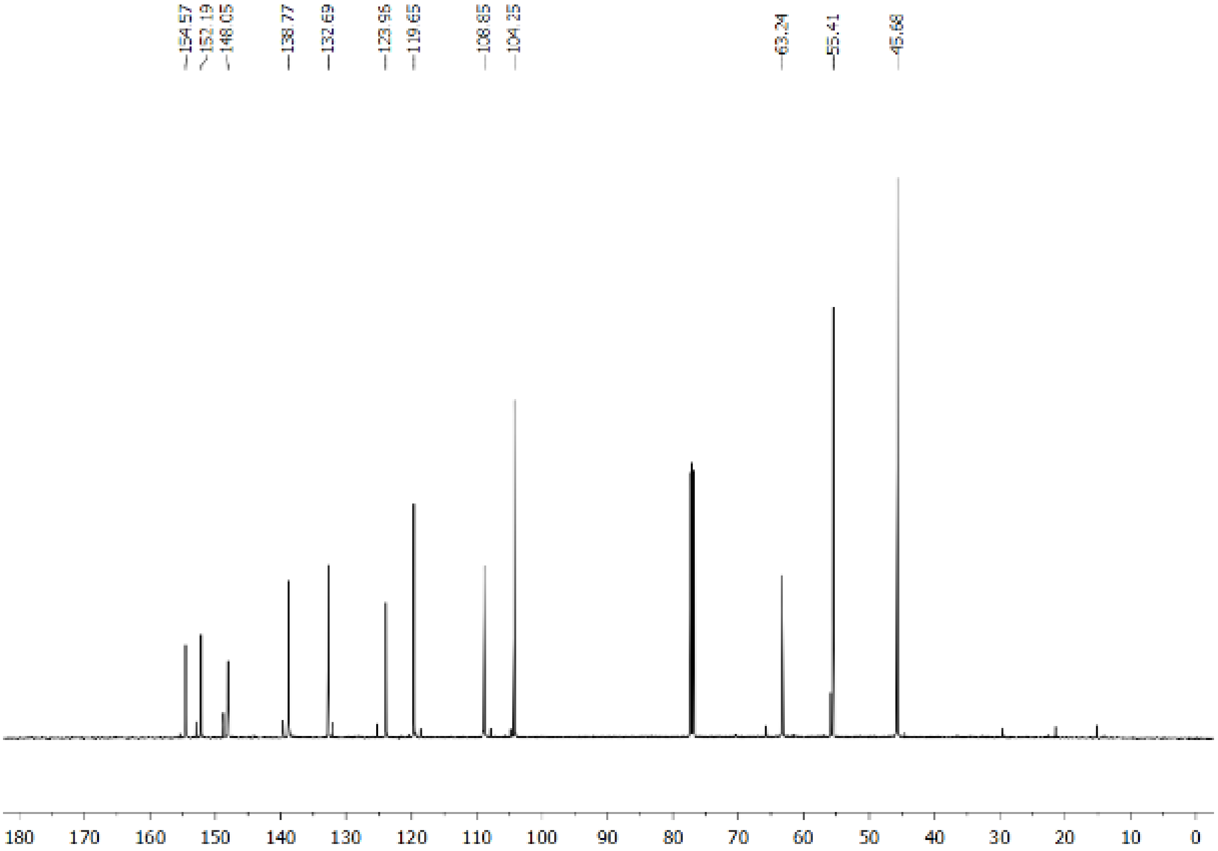

^{1}H NMR spectrum of 1-(7-benzyloxy-6-methoxyisoquinolin-1-yl)-*N,N,N*-trimethylmethanaminium iodide (7)

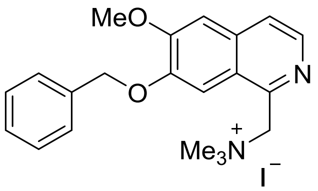

Frequency: 500 MHz

Solvent: $(CD_3)_2SO$

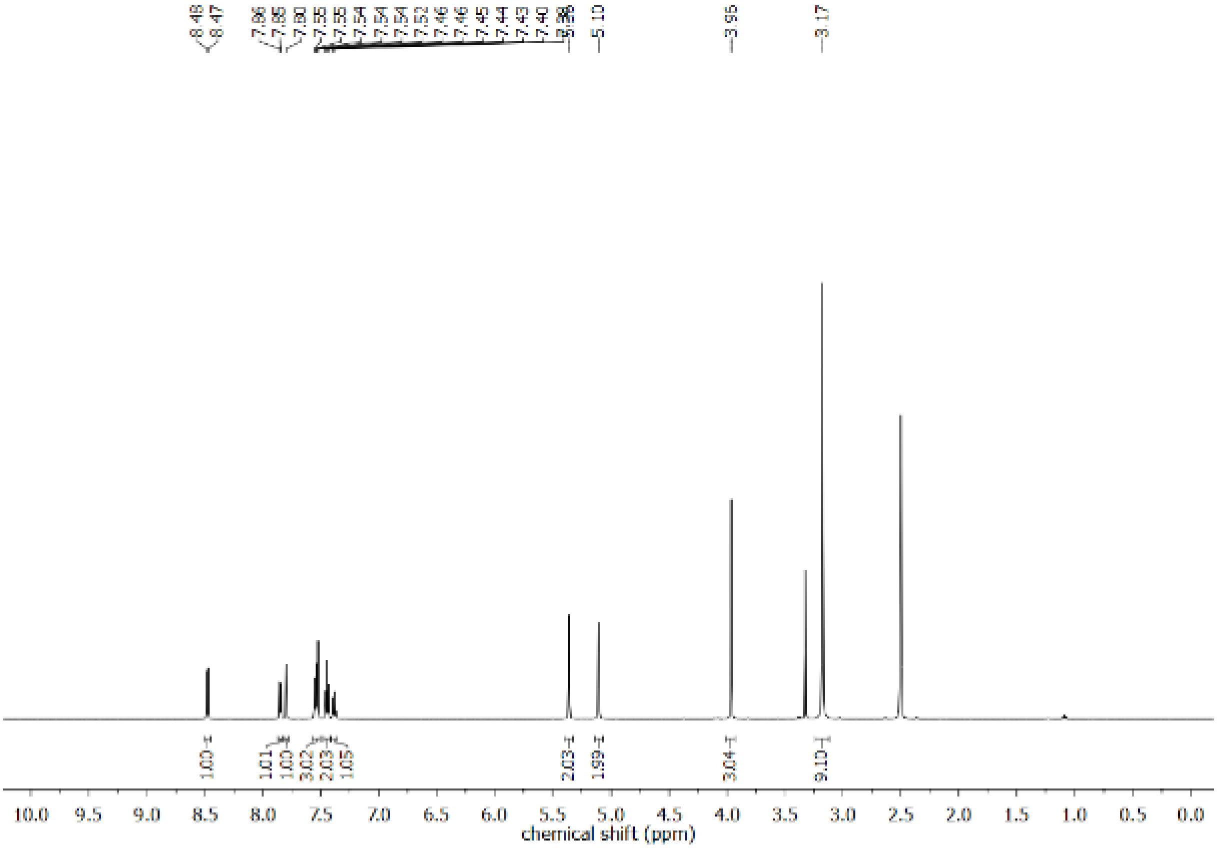

^{13}C NMR spectrum of 1-(7-benzyloxy-6-methoxyisoquinolin-1-yl)-*N*,*N*,*N*-trimethylmethanaminium iodide (7)

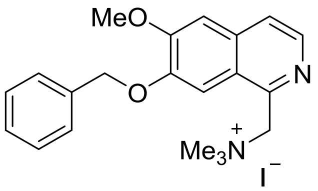

Frequency: 126 MHz

Solvent: $(CD_3)_2SO$

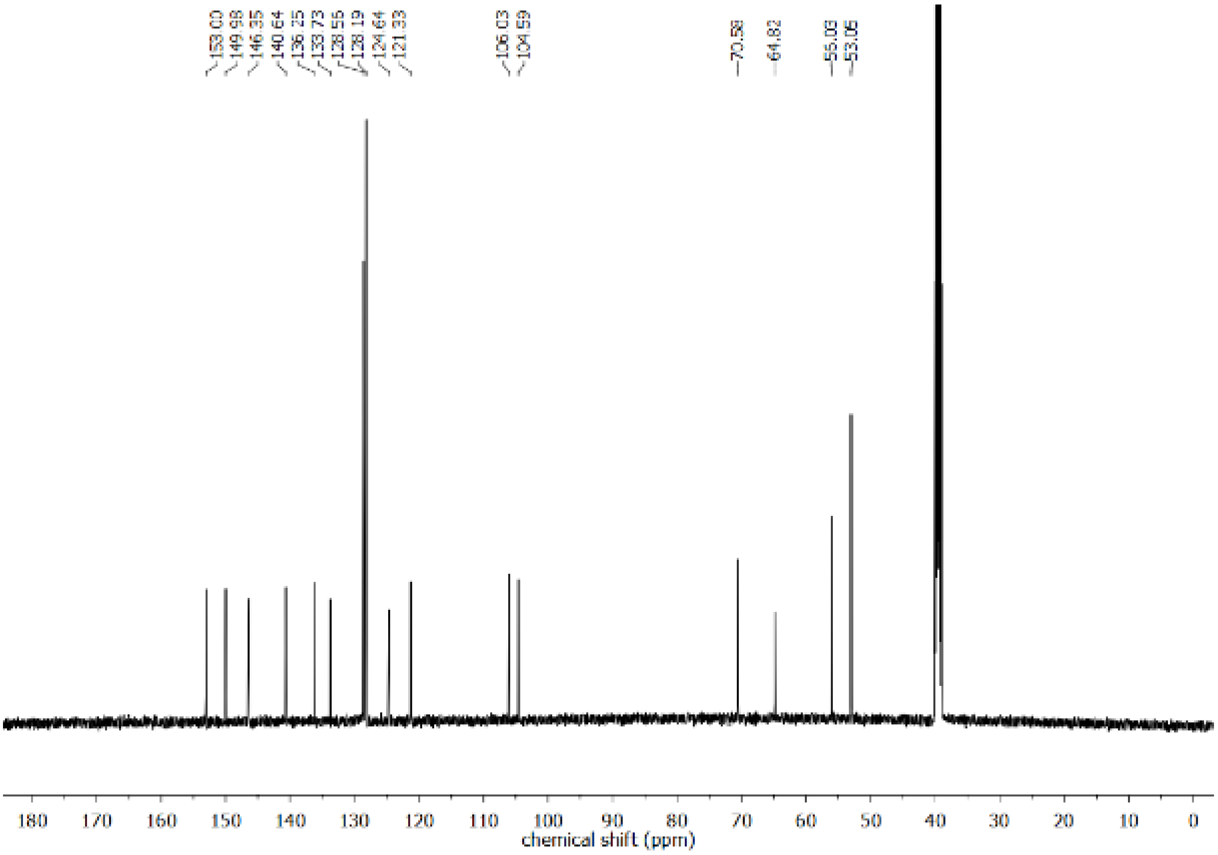

^{1}H NMR spectrum of 6-methoxy-1-methylisoquinolin-7-ol (1)

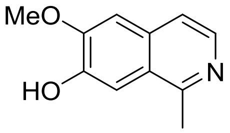

Frequency: 400 MHz

Solvent: $CDCl_3$

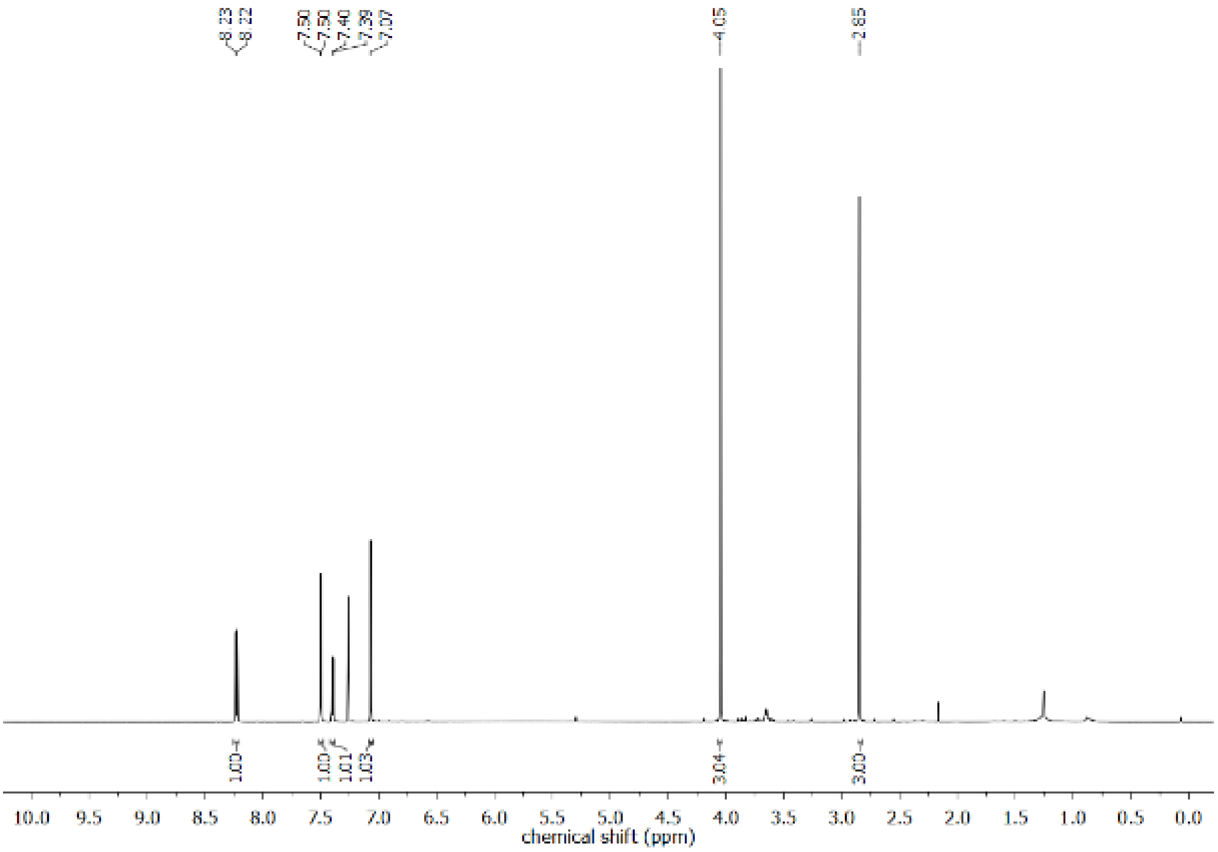

^{13}C NMR spectrum of 6-methoxy-1-methylisoquinolin-7-ol (1)

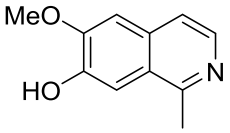

Frequency: 126 MHz

Solvent: $CDCl_3$

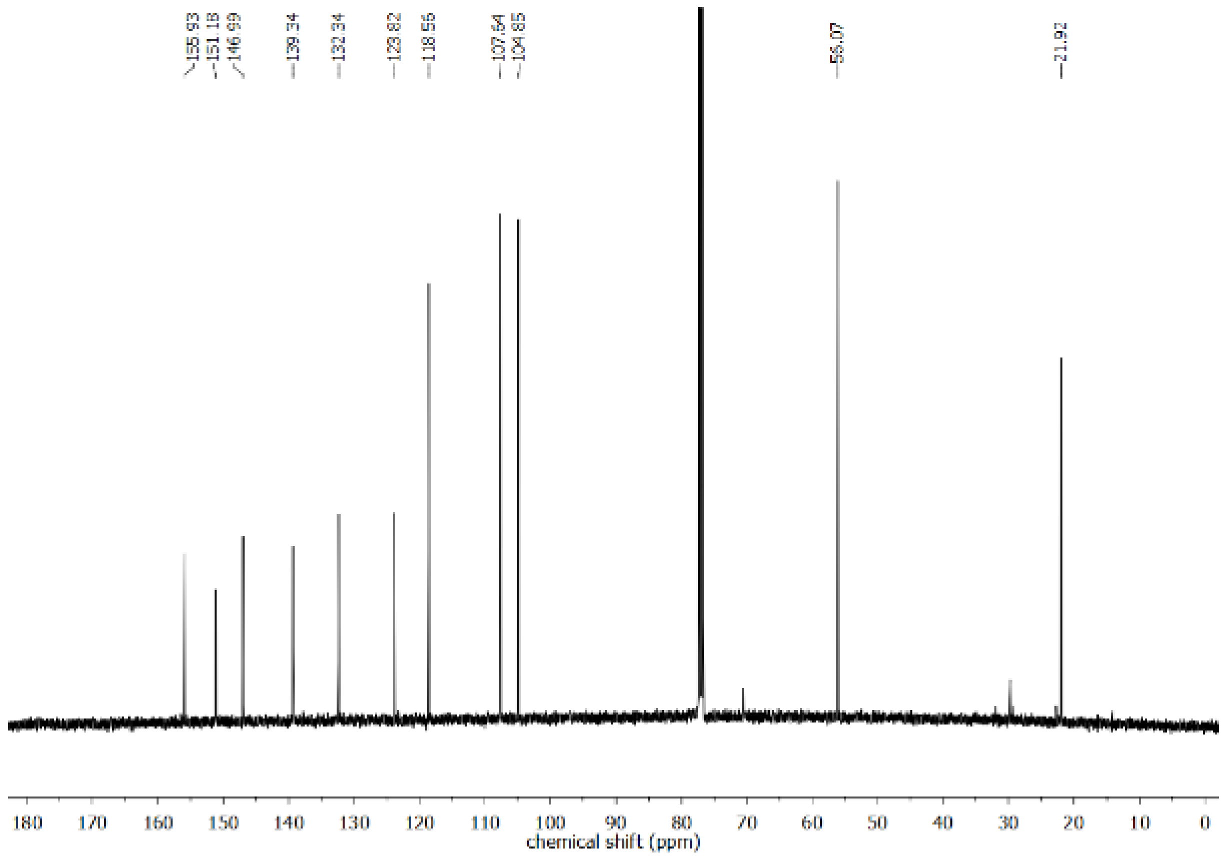

References

1. Suess, T. R.; Stermitz, F. R., *J. Nat. Prod.* **1981**, *44*, 688–692.
2. Burchard, F.; Blaschke, G. *Liebigs Ann. Chem.* **1963**, *668*, 145–164.
3. Bruderer, H.; Brossi, A. *Helv. Chim. Acta* **1965**, *48*, 1945–1956.
